# 566 Covariate Analysis of Performance of Multi-Spectral Imaging System Augmented with Artificial Intelligence

**DOI:** 10.1093/jbcr/iraf019.195

**Published:** 2025-04-01

**Authors:** Jeffrey Carter, Jeffrey Shupp, Herbert Phelan, James Hwang, Alisa Savetamal, Steven Wolf, Michael DiMaio, Kathleen Romanowski, Arpana Jain, Kevin Foster, Steven Kahn, James Holmes

**Affiliations:** Louisiana State University Burn Center; MedStar Washington Hospital Center; Louisiana State University Burn Center; UAB; Yale University; University of Texas Medical Branch; Baylor Health; University of California Davis; Valleywise Maricopa Burn Center; Diane & Bruce Halle Arizona Burn Center; Medical University of South Carolina; Wake Forest University

## Abstract

**Introduction:**

Burn wound assessment is essential for effective clinical management and prompt intervention. Typically, burn assessments rely on the clinician’s judgment to discriminate superficial partial-thickness (“healing”) from deep partial- and full-thickness (“non-healing”) burn areas. These assessments are subjective, leading to variability in diagnosis and treatment plans. This study investigates potentially confounding covariates for the performance metric of sensitivity of a non-invasive, non-contact, multispectral imaging system augmented with an artificial intelligence (AI)-trained algorithm for differentiating non-healing areas within burn wounds.

**Methods:**

In a multi-center, IRB-approved study (NCT05023135), a multispectral imaging device was used to image subjects with thermal burn injuries at 11 burn centers. Subjects were enrolled and imaged within 72 hours of injury, then serially imaged until seven days post-injury. Device AI-outputs were not presented to clinical staff and, at the discretion of the attending surgeon, burn wounds were either allowed to heal spontaneously or surgically treated. After follow-up, the algorithm was trained using the ‘ground truth’ as determined by an independent panel developed a consensus determination of the true non-healing burn areas within each multispectral image using a standardized histologic algorithm incorporating burn wound biopsies or 21-day healing assessments. Using multispectral images and the ground truth, an ensemble of 19 convolutional neural networks (CNNs) were trained to identify non-healing burn tissue within the wound. Performance of the ensemble of CNNs was calculated using cross-validation, and covariate analysis was conducted to assess the influence of patient-specific factors on sensitivity.

**Results:**

The covariates of age, gender, race, and Fitzpatrick Skin Tone were investigated as potentially impacting AI-model sensitivity. Statistical analysis using a backward elimination method with a significance level of 0.01 to create a reduced model did not find any significant association with demographic variables and sensitivity.

**Conclusions:**

Covariate analysis highlights the importance of considering patient factors in optimizing this technology’s application to ensure accurate interpretation of burn wound images. Future research will examine the impact of covariates on other performance metrics, including accuracy and specificity.

**Applicability of Research to Practice:**

This research enhances applicability by controlling confounding factors, ensuring more accurate results to bridge clinical research and real-world practice.

**Funding for the Study:**

This project is being supported in whole or in part with federal funds from the Department of Health and Human Services; Administration for Strategic Preparedness and Response; BARDA, under contract number 75A50123C00049. The findings and conclusions have not been formally disseminated by the Department of Health and Human Services and should not be construed to represent any agency determination or policy.

